# Does Enhanced External Counterpulsation (EECP) Significantly Affect Myocardial Perfusion?: A Systematic Review & Meta-Analysis

**DOI:** 10.1371/journal.pone.0151822

**Published:** 2016-04-05

**Authors:** Xiaoxia Qin, Yanye Deng, Dandong Wu, Lehua Yu, Rongzhong Huang

**Affiliations:** Department of Rehabilitation Medicine, the Second Affiliated Hospital of Chongqing Medical University, Chongqing, China; Kurume University School of Medicine, JAPAN

## Abstract

**Background:**

Enhanced external counterpulsation (EECP) is currently applied for treating coronary artery disease (CAD) patients. However, the mechanism(s) by which EECP ameliorates angina pectoris and long-term left ventricular function remain largely unknown. The aim of this study will be to assess whether EECP significantly affects myocardial perfusion in CAD patients through a systematic review and meta-analysis of the available literature.

**Methods:**

MEDLINE, EMBASE, and Cochrane CENTRAL databases were searched for prospective studies on CAD patients that underwent EECP and reported myocardial perfusion data pre- and post-EECP. The impact of EECP was assessed based on the weighted mean difference (WMD) in myocardial perfusion from pre-EECP to post-EECP. Statistical heterogeneity was assessed by the I^2^ index. Publication bias was assessed through visual inspection of the funnel plot as well as Begg’s and Egger’s testing.

**Results:**

Standard EECP therapy (i.e., 35–36 one-hour sessions within a seven-week period) significantly increased myocardial perfusion in CAD patients (pooled WMD: -0.19, 95% CI: -0.38 to 0.00, *p* = 0.049). A random effects analysis was applied on account of significant heterogeneity (I^2^ = 89.1%, *p* = 0.000). There was no evidence of significant publication bias (Begg’s *p* = 0.091; Egger’s *p* = 0.282).

**Conclusions:**

Standard EECP therapy significantly increases myocardial perfusion in CAD patients. This study’s findings support the continued use of standard EECP therapy in CAD patients and provides one putative physiological mechanism to help explain the improvements in angina pectoris and long-term left ventricular function observed in CAD patients after EECP therapy.

## Introduction

Enhanced external counterpulsation (EECP) is an outpatient coronary artery disease (CAD) therapy that involves the cyclical inflation/deflation of cuffs wrapped around the lower extremities [1]. EECP is currently applied for treating CAD patients that have failed to adequately respond to conventional revascularization interventions and pharmacotherapy [2]. Several clinical trials have provided evidence that EECP is both safe and efficacious in ameliorating angina pectoris, long-term left ventricular function, exercise capacity, and quality of life over a period of five years post-therapy [3–6]. These results have also been validated in large-scale patient populations via the International Patient Registry (IEPR) and the EECP Clinical Consortium [7].

Despite these favorable findings supporting the clinical application of EECP in CAD patients, the mechanism(s) by which EECP ameliorates angina pectoris and long-term left ventricular function remain largely unknown. One hypothesis is that EECP promotes coronary blood flow and enhances coronary perfusion pressure [7]. However, this hypothesis remains controversial, as there is conflicting evidence from previous EECP studies. Therefore, the aim of this study was to assess whether EECP significantly affects myocardial perfusion in CAD patients through a systematic review and meta-analysis of the available literature.

## Materials and Methods

This meta-analysis was conducted according to the Preferred Reporting Items for Systematic Reviews and Meta-Analyses (PRISMA) guidelines (S1 Fig) [8].

### Search Strategy

We searched MEDLINE, EMBASE, and Cochrane CENTRAL databases from inception until May 2015 for relevant studies using the following key search terms: (“enhanced external counterpulsation” OR EECP) AND perfusion. In addition, a manual search of relevant review articles were screened for additional records. The literature search was limited to human clinical trials reported in English.

### Selection Criteria

Two authors independently selected studies for inclusion with disagreements resolved by discussion and consensus. Studies were included for meta-analysis if they had a prospective design, included patients with a diagnosis of CAD that underwent EECP, and reported myocardial perfusion data pre- and post-EECP. According to patient selection criteria elaborated by early EECP research [9], CAD was defined as one of the following: (i) coronary luminal stenosis (>70%) in at least one major coronary artery by angiography, (ii) enzymatic and/or electrocardiographic (ECG) evidence of myocardial infarction (MI), or (iii) a positive nuclear exercise stress test for MI or ischemia. As the EECP procedure makes the performance of controlled trials challenging, all types of prospective studies were included here. Retrospective studies, non-human studies, case reports/series, conference abstracts/summaries, and reviews were excluded.

### Data Extraction

The following data were independently extracted from the studies by two authors through the use of a standardized data abstraction sheet with disagreements resolved by discussion and consensus: first author’s name, year of publication, country of study, study design, patient selection criteria, study sample size, protocol and duration of EECP therapy, method of measuring myocardial perfusion and/or intracoronary pressure, data collection time points (pre- and post-EECP), and reported data on changes from baseline in one of two outcomes: (i) myocardial perfusion or coronary flow and/or (ii) intracoronary pressure.

### Quality Assessment

Two authors independently assessed study quality with disagreements resolved by discussion and consensus. The methodological quality of the included studies was evaluated using the Newcastle-Ottawa scale (NOS) for prospective cohort studies [10]. Study quality was assessed on the selection of study groups, comparability of study groups, and ascertainment of outcomes with a maximum score of nine points. Studies scoring below five points were deemed to be of low-quality, those scoring five to seven points were deemed to be moderate quality, and those scoring eight or nine points were deemed to be of high-quality.

### Statistical Analysis

Based on the available evidence, only one outcome—myocardial perfusion or coronary flow–was amenable to meta-analysis. All statistical analyses were performed using RevMan 5.0 (Cochrane Collaboration). Statistical heterogeneity was assessed by the I^2^ index with significant heterogeneity defined as an I^2^ value of greater than 50% and a *p*-value of less than 0.05 [11]. A random-effects model was used in the presence of significant heterogeneity [12]. The impact of EECP was assessed based on the weighted mean difference (WMD) in myocardial perfusion from pre-EECP to post-EECP. Publication bias was assessed through visual inspection of the funnel plot as well as Begg’s and Egger’s testing [13].

## Results

**Fig 1:**
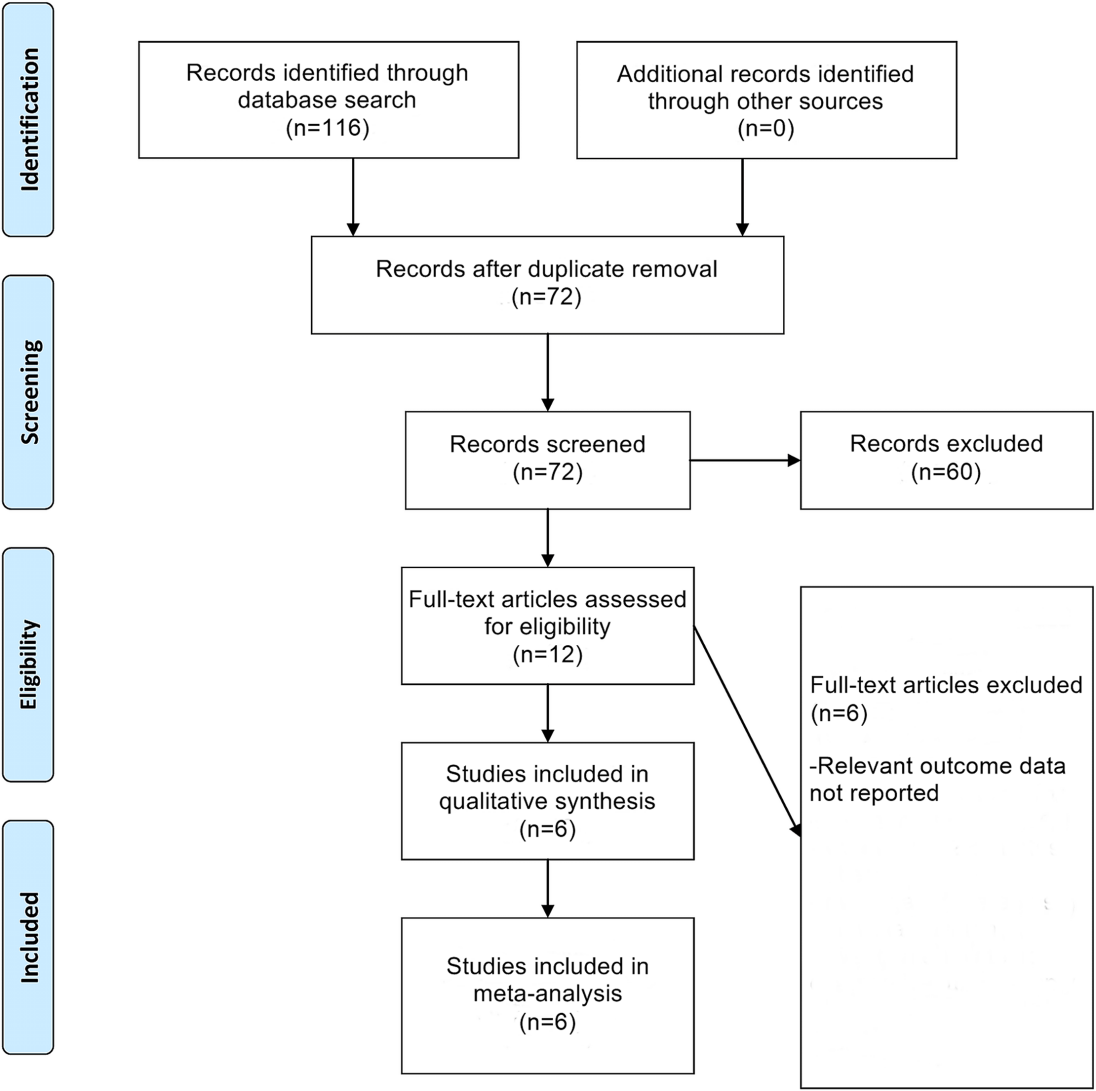
Flowchart of Study Selection.

The flowchart of study selection is provided in Fig 1. From an initial set of 72 non-duplicate records, six studies were finally included in this meta-analysis after application of the inclusion and exclusion criteria [14–19]. The characteristics of these six included studies are provided in Table 1. The NOS quality assessment of these studies is detailed in Table 2. All the included studies received high-quality NOS scores.

**Table 1:** Characteristics of Included Studies.

Study	Country	Design	Selection criteria	Sample size (n)	Protocol and duration of EECP therapy	Technique of measuring myocardial perfusion	Data collection time points (pre- and post-EECP)
Arora 2007	USA	Prospective, randomized, blinded	MUST-EECP inclusion and exclusion criteria*	11	One-hour sessions (once or twice daily, five days per week) for a total of 35 hours over four-seven weeks	Dynamic ^13^N positron emission tomography (PET)	Less than four weeks pre-EECP; less than two weeks post-EECP
Lawson 1992	USA	Prospective, randomized, blinded	Included chronic, stable angina patients with exertional ischemia; excluded clinical CHF, aortic insufficiency, MI in the past three months, significant ventricular ectopic activity or atrial fibrillation, non-ischemic cardiomyopathy, severe occlusive peripheral vascular disease, recurrent deep vein thrombophlebitis, blood pressure >180/110 mm Hg, or bleeding diathesis	18	One-hour daily sessions for a total of 36 hours	Thallium-201 scan	Immediately before EECP; within one week post-EECP
Masuda 2001	Japan	Prospective, observational, blinded	Included CAD patients (aged 21–81) with CCSC I-III; MUST-EECP exclusion criteria*	11	One-hour sessions (once or twice daily) for a total of 35 hours over a period of 18–35 days	Dynamic ^13^N positron emission tomography (PET)	Immediately before and after EECP
Michaels 2002	USA	Prospective, randomized, blinded	Included CAD outpatients referred for catheterization; excluded severe aortic insufficiency, decompensated CHF, significant arrhythmia, systolic blood pressure >180 mm Hg, symptomatic PVD, abnormal Doppler Allen’s test of right upper extremity, or unsuitable lower extremity/coronary anatomy	10	EECP protocol not reported	0.014” Doppler velocity guidewire positioned in the mid-to-distal portion of an unobstructed coronary artery under fluoroscopic guidance	Immediately before and during EECP
Michaels 2005	USA	Prospective, randomized, blinded	Included CAD patients with CCSC II-IV or angina averaging at least twice weekly; MUST-EECP exclusion criteria*	34	One-hour sessions (once daily, five days per week) for a total of 35 hours over a seven-week period	Quantitative gated technetium Tc 99m sestamibi single photon emission computed tomography (SPECT) exercise perfusion imaging	Immediately before EECP; one month post-EECP
Tartaglia 2003	USA	Prospective, randomized, blinded	Included CAD patients with CCSC class II or higher; excluded if unable to treadmill, MI within past six weeks, unstable angina, uncontrolled hypertension, severe valvular heart disease, or malignant ventricular arrhythmia	25	One-hour sessions (once or twice daily) for a total of 35 hours	Single-photon emission computed tomography (SPECT)	Immediately before and after EECP

*Multicenter study of EECP (MUST-EECP) inclusion criteria: 23–82 years of age; Canadian Cardiovascular Society Class (CCSC) I, II, or III; coronary artery disease (CAD) evidenced by one of three measures; angiographic-proven disease of one major coronary artery, enzymatic and/or electrocardiographic (ECG) evidence of myocardial ischemia (MI), or positive nuclear exercise stress test for MI or ischemia.

MUST-EECP exclusion criteria: pregnant or of child-bearing potential and not using a contraceptive method; presenting with unstable angina, arrhythmias, or marked ECG abnormalities; MI or coronary artery bypass grafting (CABG) in the past three months; cardiac catheterization in the past two weeks; permanent pacemaker or implantable defibrillator; currently enrolled in a cardiac rehabilitation program; or any of the following: overt cardiac heart failure (CHF, left ventricular ejection fraction less than 30%); significant valvular disease; severe symptomatic peripheral vascular disease, history of varicosities, deep vein thrombosis/pulmonary embolism, phlebitis, and/or stasis ulcer; uncontrolled hypertension (greater than 180/110 mm Hg); bleeding diathesis; warfarin use with international normalized ratio of greater than 2.0; unable to treadmill test; or non-bypassed left main disease of greater than 50%.

**Table 2:** Newcastle-Ottawa Scale Quality Assessment of Included Studies.

Study	Is the case definition adequate? (one point)	Representativeness of the cases (one point)	Selection of controls (one point)	Definition of controls (one point)	Comparability of cases and controls on the basis of the design or analysis (two points)	Assessment of outcome (one point)	Was follow-up long enough for outcomes to occur? (one point)	Adequacy of cohort follow-up (one point)	Total score (nine points)
Arora 2007	1	1	1	1	2	1	1	1	9
Lawson 1992	1	1	1	1	2	1	1	1	9
Masuda 2001	1	1	1	1	2	1	1	1	9
Michaels 2002	1	1	1	1	2	1	0	1	8
Michaels 2005	1	1	1	1	2	1	1	1	9
Tartaglia 2003	1	1	1	1	2	1	1	1	9

**Fig 2:**
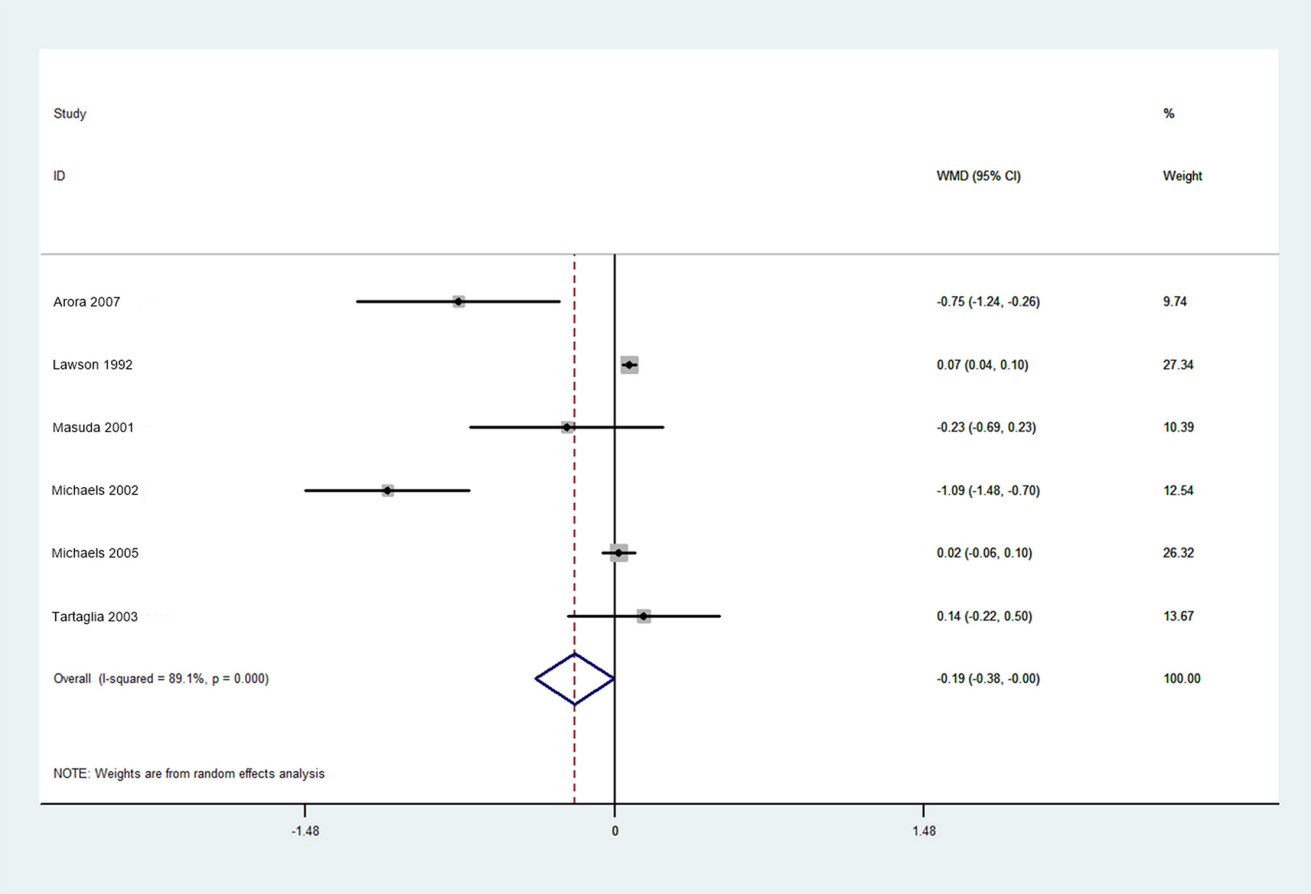
Forest Plot of Weighted Mean Differences in Myocardial Perfusion Pre- and Post-EECP.

**Fig 3:**
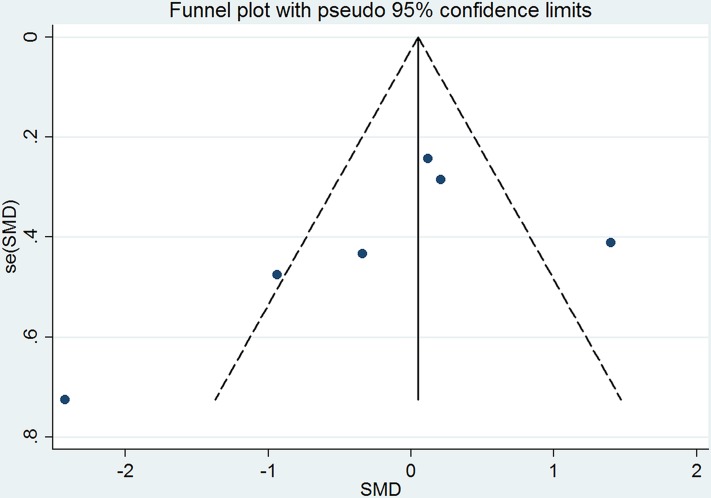
Funnel Plot of Included Studies.

Based on the available evidence, only one outcome—myocardial perfusion or coronary flow–was amenable to meta-analysis. The pre- and post-EECP myocardial perfusion values from the included studies are listed in Table 3. We found that standard EECP therapy (i.e., 35–36 one-hour sessions within a seven-week period) significantly increased myocardial perfusion in CAD patients (pooled WMD: -0.19, 95% CI: -0.38 to 0.00, *p* = 0.049; Fig 2). A random effects analysis was applied on account of significant heterogeneity (I^2^ = 89.1%, *p* = 0.000; Fig 2). Although visual inspection of the funnel plot appeared to reveal publication bias (Fig 3), there was no statistically significant publication bias detected by either Begg’s and Egger’s testing (Begg’s *p* = 0.091; Egger’s *p* = 0.282).

**Table 3:** Myocardial Perfusion Results from Included Studies.

Study	Pre-EECP data			Post-EECP data		
	Sample size (n)	Mean	Standard deviation	Sample size (n)	Mean	Standard deviation
Arora 2007	11	1.00	0.20	11	1.75	0.80
Lawson 1992	18	1.00	0.05	18	0.93	0.05
Masuda 2001	11	1.00	0.39	11	1.23	0.68
Michaels 2002	10	1.00	0.45	10	2.09	0.45
Michaels 2005	34	1.00	0.16	34	0.98	0.17
Tartaglia 2003	25	1.00	0.64	25	0.86	0.67

## Discussion

Here, we assessed whether EECP significantly affects myocardial perfusion in CAD patients through a systematic review and meta-analysis of the available literature. We found that standard EECP therapy (i.e., 35–36 one-hour sessions within a seven-week period) significantly increased myocardial perfusion in CAD patients. This study’s findings support the continued use of standard EECP therapy in CAD patients and provides one putative physiological mechanism to help explain the improvements in angina pectoris and long-term left ventricular function observed in CAD patients after EECP therapy.

Standard EECP therapy is based on a biomechanical device consisting of three basic components: a set of cuffs, an air compressor/pump, and a computer system [17]. Initially, the set of cuffs are wrapped around the calves, lower thighs, upper thighs, and buttocks bilaterally [17]. The cuffs are then attached to the air compressor by hoses, which allows the cuffs to be cyclically inflated and deflated in synchrony with the patient’s cardiac cycle. Beginning in early diastole, pressure (100–300 mm Hg) is sequentially applied in the cranial direction from the calves to the buttocks [17]; this sequential pressure wave produces retrograde aortic flow that enhances coronary artery mean pressure by 16% and peak diastolic pressure by 93% [20]. Then, the pressurized air is quickly exhausted from the cuffs at the completion of diastole [17]. The foregoing cycle is repeated over the course of a one-hour session; these one-hour sessions are typically performed 35–36 times within the course of seven weeks. Notably, all of the studies included in this meta-analysis (with the exception of Michaels 2002 [17], which failed to report on the precise EECP protocol used) applied this standard EECP protocol.

**Fig 4:**
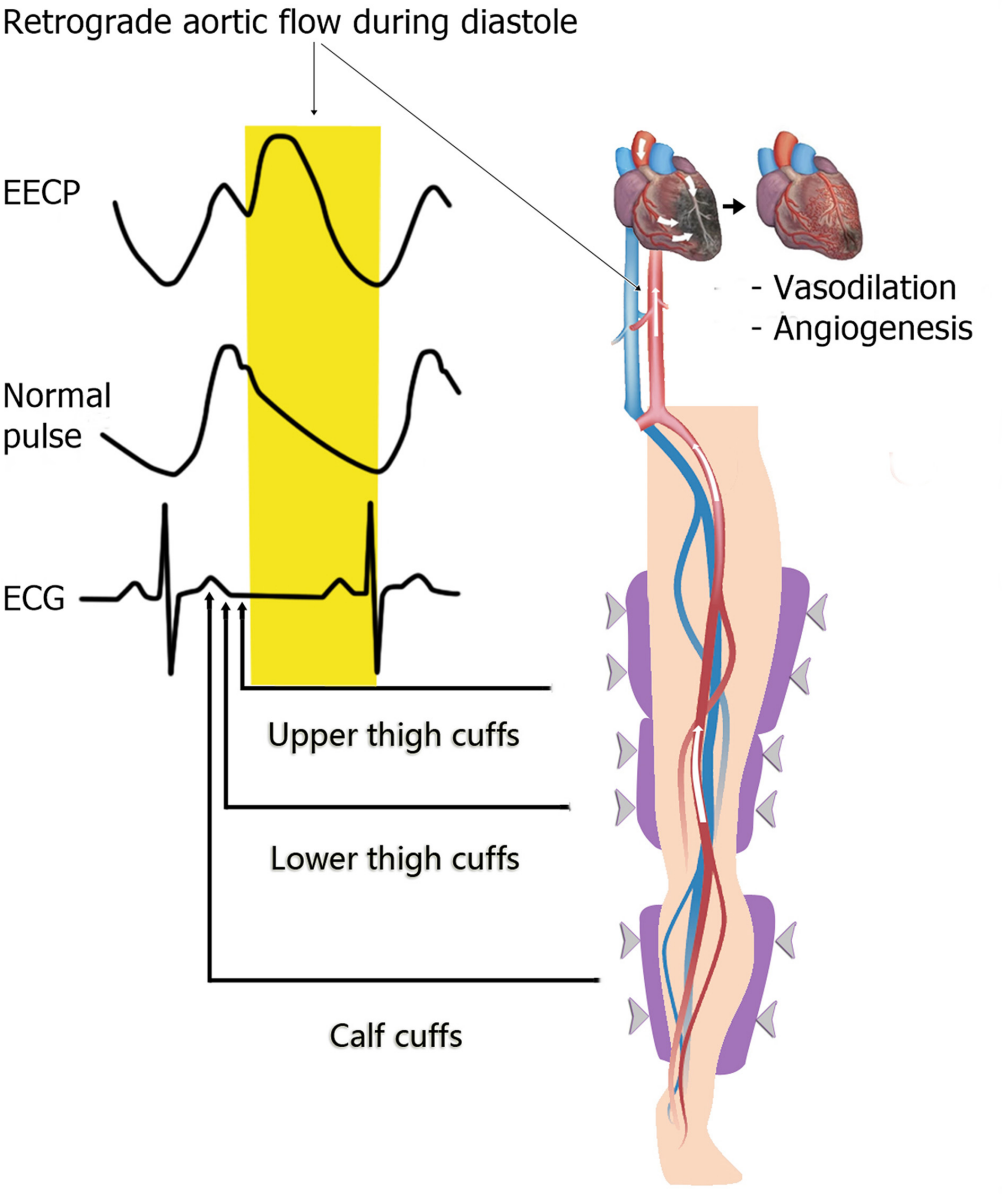
Putative Physiological Mechanisms underlying EECP’s Effects on Myocardial Perfusion. EECP produces a diastolic retrograde aortic flow that enhances coronary artery mean pressure and peak diastolic pressure. This retrograde aortic flow acts to improve coronary vasodilation and angiogenesis through three putative mechanisms. First, through its pressure wave, EECP directly vasodilates existent vessels in the myocardium. Second, EECP increases de novo collateral vessel formation through promoting the release of angiogenic and vasoactive factors such as α-actin, von Willebrand factor (vWF), vascular endothelial growth factor (VEGF), basic fibroblast growth factor (BFGF), and hepatocyte growth factor (HGF). Third, EECP increases shear stress in the vasculature, a process which promotes the release of the angiogenic vasodilator nitric oxide (NO).

With specific respect to myocardial perfusion, our findings validate Michaels 2002’s Doppler and angiographic data that showed a 150% increase in coronary flow velocity as well as a 28% increase in coronary flow post-EECP [17, 20]. Mechanistically, EECP appears to enhance myocardial perfusion through improving coronary vasodilation and angiogenesis (Fig 4) [20]. One hypothesis is that the EECP pressure wave directly dilates existent vessels in the myocardium [20]. Another hypothesis–directly supported by Wu et al.’s work in canines—is that EECP increases de novo collateral vessel formation in the myocardium through promoting the release of angiogenic and vasoactive factors such as α-actin, von Willebrand factor (vWF), and vascular endothelial growth factor (VEGF) [20, 21]. Moreover, Masuda et al. has also shown that EECP upregulates other angiogenic factors, including basic fibroblast growth factor (BFGF) and hepatocyte growth factor (HGF) [22]. In addition, EECP increases shear stress in the vasculature, a process which promotes the release of the angiogenic vasodilator nitric oxide (NO) [20, 23, 24]. These hypotheses support our current findings that show a significant improvement in myocardial perfusion in CAD patients following EECP.

There are several limitations to this study. First, the study sample size of this meta-analysis was rather limited; thus, more clinical trials that measure myocardial perfusion (as well as intracoronary pressure) before and after EECP are needed. Second, the studies included in this meta-analysis employed a variety of methods for measuring myocardial perfusion, which may have affected the results. Third, one included study (Michaels 2002 [17]) compared pre-EECP myocardial perfusion against myocardial perfusion during EECP as opposed to post-EECP; therefore, inclusion of this study may have adversely affected the analysis. Fourth, there was a significant level of heterogeneity detected in this meta-analysis. On the basis of these limitations, future clinical studies on EECP should aim to (i) employ a prospective design, randomization, and blinding, (ii) recruit large cohorts of CAD patients to ensure adequate powering, (iii) diligently record relevant demographic and clinical factors that may affect the efficacy of EECP (e.g., body mass index (BMI), post-coronary artery bypass grafting (CABG) status, peripheral vascular disease (PVD) status, smoking status, etc.) and segregate patients into sub-cohorts based on these factors to allow factor-based analysis, (iv) employ the standardized EECP protocol (i.e., 35–36 one-hour sessions within a seven-week period) using gold-standard equipment to ensure broad applicability of the findings, (v) employ multiple techniques to assess myocardial perfusion to enable data cross-checking across techniques, (vi) compare pre-EECP myocardial perfusion against post-EECP myocardial perfusion (i.e., immediately before and after EECP) in order to assess the full effects of EECP therapy, and (vii) ensure a sufficient follow-up period (e.g., three months, six months, or one year post-EECP) to enable examination of the sustainability of EECP’s effects.

In conclusion, standard EECP therapy significantly increases myocardial perfusion in CAD patients. This study’s findings support the continued use of standard EECP therapy in CAD patients and provides one possible physiological mechanism to help explain the improvements in angina pectoris and long-term left ventricular function observed in CAD patients after EECP therapy.

## Supporting Information

S1 Fig Preferred Reporting Items for Systematic Reviews and Meta-Analyses (PRISMA) Checklist. (DOC)
